# Temperature and Time Dependence of the Solvent-Induced Crystallization of Poly(*l*-lactide)

**DOI:** 10.3390/polym12051065

**Published:** 2020-05-06

**Authors:** Mahitha Udayakumar, Mariann Kollár, Ferenc Kristály, Máté Leskó, Tamás Szabó, Kálmán Marossy, Ildikó Tasnádi, Zoltán Németh

**Affiliations:** 1 Higher Education and Industry Cooperation Centre of Advanced Materials and Intelligent Technologies, University of Miskolc, H-3515 Miskolc, Hungary; kemudaya@uni-miskolc.hu; 2 Institute of Chemistry, University of Miskolc, H-3515 Miskolc, Hungary; 3 Institute of Ceramic and Polymer Engineering, University of Miskolc, H-3515 Miskolc, Hungary; femmaja@uni-miskolc.hu (M.K.); polsztam@uni-miskolc.hu (T.S.); polkal01@uni-miskolc.hu (K.M.); poltildi@uni-miskolc.hu (I.T.); 4 Institute of Mineralogy and Geology, University of Miskolc, H-3515 Miskolc, Hungary; askkf@uni-miskolc.hu (F.K.); askmate@uni-miskolc.hu (M.L.)

**Keywords:** poly(*l*-lactide), solvent-induced crystallization, swelling, solubility parameter, temperature and time dependence

## Abstract

The role of organic solvents in governing the crystallization and morphology of semi-crystalline poly-*l*-lactide (PLLA) sheets was systematically investigated. Three different organic solvents; ethyl acetate (EA), o-dichlorobenzene (ODCB), and nitrobenzene (NB), with a solubility parameter analogous to PLLA and with a high capability of swelling, were chosen. It has been witnessed that the degree of crystallization and crystal morphology depends highly on the degree of swelling and evaporation rate of the solvent. Besides, the temperature and time of treatment played a significant role in the crystallization of polymers. The effect of different solvents and curing times are reflected by the measured X-ray diffraction (XRD) peaks and the differences are best shown by the unit cell size. The largest variation is observed along the c-axis, indicating shorter bonds, thus, showing better conformation after NB and ODCB treatment. The percentage of crystallinity calculated using the classical relative crystallinity index of XRD shows closer values to those calculated with differential scanning calorimetry (DSC) data, but a huge variation is observed while using the LeBail deconvolution method. The strong birefringence of polarised optical micrograph (POM) and the crystal morphology of scanning electron micrograph (SEM) also evidenced the orientation of polymer crystallites and increased crystallinity after solvent-supported heat treatment.

## 1. Introduction

Due to the depletion of fossil fuels and the huge environmental impact of extraction methods, the production of plastics from such fossil fuels is not a sustainable way to do so [1], and furthermore, their wastes, discarded into the environment, are subject to slower degradation. Biodegradable polymers are the potential replacement for petroleum-based polymers and other bio-compatible materials, such as metals and ceramics [2]. The most widely using biodegradable polymers are polylactide (PLA), polycaprolactone (PCL), poly(butylene adipate terephthalate) (PBAT) and polyhydroxybutyrate (PHB) [3]. Among these, PLA is one of the major commercial biopolymers derived from renewable raw materials such as corn starch, potato, sugarcane and even from food wastes [4]. Nowadays, the PLA has been widely used in the packaging industries due to its biodegradability, good mechanical and translucent properties [5]. Based on its biocompatibility, special grades of PLA had been developed for biomedical applications such as drug delivery systems [6].

Generally, the PLA can be produced by two polymerization routes, direct condensation of lactic acid monomer or ring-opening polymerization of lactide dimer (*l*-, *d*- or *d*,*l*-), with various metal catalysts in the form of a solution, melt, or as a suspension [7]. There are three possible ways to produce PLA from lactides: poly(*l*-lactide) (PLLA) and poly(*d*-lactide) (PDLA) from the polymerization of *l*,*l*-lactide and *d*,*d*-lactide, respectively, and the combination of *l*- and *d*- lactides usually lead to the synthesis of poly(*d*,*l*-lactide) (PDLLA) [8]. The properties of PLA depend primarily on the stereochemistry of the lactide (*l*-, *d*- or *d*,*l*-) isomers and the thermal history (annealing) during processing [9]. 

The thermal, mechanical and other significant properties of PLA depend mainly on its degree of crystallinity [10]. Therefore, the understanding of the crystallization behaviour and kinetics of PLA is substantial to control its properties. However, the crystallization of PLA is rather a slow process compared to many conventional thermoplastics [11]. The polymer crystallization can be done by following different approaches such as physical aging [12], phase separation [13], process-induced crystallization [14], nucleating-agent induced crystallization [15], and/or solvent-induced crystallization [16]. The research about the thermal annealing and solvent-induced crystallization of the PLA has been increasingly paid attention to, not only to improve their mechanical, thermal and other properties, but also to know the interaction of this biopolymer with different media or the humid environment during application. During polymer-solvent interaction, the solvent molecules go into the polymer and increase the chain mobility and because of polymer chain segmental relaxation, crystallization occurs, even at room temperature in many polymers [17].

In the past, only limited research works have been done on the temperature dependence of solvent-induced crystallization of PLA. Iwata, T. et al. [18] reported the preparation of lozenge- and hexagonal shaped PLLA single crystal with spiral growth from a dilute solution of p-xylene and studied the enzymatic degradation of single crystal by Proteinase-K. The barrier properties of commercial PLA were studied using organic vapours of ethyl acetate and d-limonene and affirmed that the PLA is not likely to promote flavour loss [19]. Sawada, H et al. [20] tested the combined effect of heat treatment and solvent-induced crystallization of PLAs using dichloromethane and investigated their gas transport properties. Gondo, D. et al. [21] investigated the crystallization of PLA membranes using methanol and ethanol and showed the formation of the α-crystal structure of PLA in methanol and a crystallized mixture of α- and β-forms in ethanol. 

Furthermore, the crystallization behaviour of amorphous PLA while immersing in many different organic solvents like acetone, ethyl acetate, diethyl ether, tetrahydrofuran, methanol, hexane, toluene, xylene, and o-dichlorobenzene had been studied and reported that acetone was the most effective solvent to accelerate the crystallization in PLA [22]. Sato, S. et al. [23] investigated the effects of 60 different organic solvents on the properties of PLA using the Hansen solubility parameter (HSP) and reported that the hydrogen bonding parameter is most effective in the solubility of the PLA films and obtained the solvent-induced crystallization on account of the degree of swelling. Moreover, the effects of mixed solvents on the surface morphology, crystallization and properties of PLA have been reported [17,24].

In the present work, we conducted experiments to determine the degree of swelling and the solubility parameter (SP) of commercial PLLA using different organic solvents. The swelling behaviour plays a significant role in inducing crystallinity during polymer-solvent interaction. Besides, the temperature and time of solvent induction are also the crucial parameters which influence the degree of crystallization in polymers. Therefore, we investigated the temperature and time dependence of solvent-induced crystallization of PLLA using three thermodynamically compatible organic solvents, generating a high degree of swelling in PLLA. This research work contributes to the better understanding of the influence of solubility parameter of solvents, and the temperature and time of solvent-induction in swelling and solvent-induced crystallization of the PLLA. 

## 2. Materials and Methods

### 2.1. Materials

Poly(*l*-lactide) sheets of 1 mm thickness, with average molecular weight (M_W_) of 2.1 × 10^5^ g/mol were purchased from Sigma-Aldrich and used as received. Organic solvents such as dichloromethane, dimethylformamide (DMF), dimethylsulfoxide (DMSO), ethyl acetate (EA), nitrobenzene (NB), nitromethane, o-dichlorobenzene (ODCB), toluene, trichloromethane (all solvents are anhydrous with ~99.9 % purity) were purchased from Sigma-Aldrich and used without further purification. MilliQ water (18.2 MΩcm) was used for the swelling experiment. 

### 2.2. Solubility Test

To investigate the solubility and the swelling behaviour of PLLA in the fore-mentioned organic solvents, the PLLA sheets, cut into 1.5 cm × 1.0 cm × 0.1 cm, were immersed into the individual solvents taken in a petri dish. In each solvent, three PLLA sheets with the same dimensions were taken to check the reproducibility of the results. The samples were steeped into the solvents for 60 min. Meanwhile, every 5 min, the samples were taken out from the petri dish, wiped them with paper tissues and weighed. The PLLA sheets in the solvents dichloromethane, trichloromethane and nitromethane started to dissolve immediately after immersion and completely dissolved within 10 to 15 min. On the other hand, the solvents such as DMF, DMSO, EA, NB, ODCB, toluene and water gets diffused into the PLLA polymeric network and made them swell. The solvent intake or degree of swelling was determined for every 5 min. The degree of swelling (%) was calculated using the following equation:

(1) $$\mathrm{Degree}\;\mathrm{of}\;\mathrm{swelling}\;(\%)=\frac{W_{F}-W_{I}}{W_{I}}\times 100$$

where *W*_I_ (g) and *W*_F_ (g) are the initial (dry) and final weight (swollen) of the samples before and after immersion, respectively. 

### 2.3. Determination of Solubility Parameter of PLLA

Two different methods were used to find the solubility parameter of the PLLA sheet and their results were compared. At first, the solubility parameter of PLLA was determined using the degree of swelling (%) of PLLA in the respective organic solvents (measured from the solubility test) and the solubility parameter of the real solvents taken from the literature [25,26]. In the second approach, the solubility parameter of PLLA was calculated theoretically by Small’s method [27] using the following equation:

δ = (ΣF)/V = (ΣF)ρ/M(2)

where δ is the solubility parameter (cal^1/2^cm^−3/2^), F is the molar attraction constants (taken from [28]); V is the molar volume; M is the molar mass of repeating unit; ρ is the density of the polymer. 1 cal^1/2^cm^−3/2^ = 2.045 MPa^1/2^.

### 2.4. Crystallization of PLLA 

**Figure 1:**
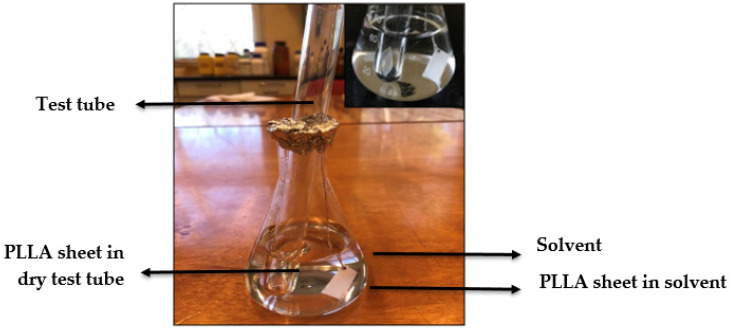
Experimental set-up for thermal annealing and solvent-induced crystallization of poly-*l*-lactide (PLLA).

The experiment for the crystallization of PLLA by thermal annealing and solvent addition was performed in a laboratory oven. The solvents nitrobenzene, o-dichlorobenzene and ethyl acetate, which strongly swelled the PLLA, were taken to study the effect of the solvents in PLLA sheets at different temperatures. The experiment was conducted in a 100 mL conical flask and a test tube. Two PLLA sheets cut into 1.5 cm × 1.0 cm × 0.1 cm were taken for each analysis. One of the sheets connected to the aluminium metal wire was immersed into the solvent taken in a conical flask and another sheet taken in a dry test tube (without any solvent) was placed inside the conical flask. This set-up (Figure 1) was adopted to make sure that the two samples were placed at the same temperature inside the oven. The experimentation was carried out at different temperatures, for ethyl acetate (50 °C and 70 °C), and o-dichlorobenzene and nitrobenzene (80 °C and 100 °C) based on their boiling points, and for a duration of 5 and 20 min. The whole set-up was covered with aluminium foil to avoid the evaporation of the solvent. After keeping the samples at the desired temperature and time, they were taken out, blotted with paper tissues and dried in air at room temperature overnight to remove the excess solvents. 

## 3. Characterization

The thermal analysis of the PLLA samples was measured with a DSC131 evo differential scanning calorimeter. The differential scanning calorimetry (DSC) sample pan-kit was aluminium. The heat scans were performed from 20 to 200 °C at a heating rate of 10 °C/min, under a nitrogen atmosphere. The glass transition temperature (*T*_g_) was determined as the midpoint of the endothermic transition. The crystallization temperature (*T*_c_) and the melting temperature (*T_m_*) were determined as the maximum of each peak. The degree of crystallinity (%X_C-DSC_) was calculated using the following equation:

(3) $${\%X}_{C-\mathrm{DSC}}=\frac{\Delta H_{m}-\Delta H_{c}}{93.1}\times 100$$

where Δ*H*_m_ and Δ*H*_c_ are the melting and crystallization enthalpies of a polymer in J/g, respectively, and 93.1 J/g is the enthalpy of the fully crystalline PLA (*l*-donor 100%) sample [29].

X-ray powder diffraction measurements (XRD) were run on a Bruker D8 Discover instrument (Cu K-alpha, 40 kV and 40 mA) in parallel beam geometry obtained with Göbel mirror, using 0.2° equatorial Soller-slit and LynxEye X-ET energy-dispersive detector in 0D mode. Patterns were recorded in the 2°–70° (2*θ*) angular region with 0.007° (2*θ*)/124 sec counting time, corresponding to regular scintillation detector counting time. Recorded patterns were evaluated by combining the LeBail–Pawley fitting in TOPAS4 software, using α-PLLA symmetry and unit cell data from [30], with instrumental convolution determined on NIST SRM 640d Si powder and using 1st-degree Tschebyshev polynomial background also determined on the Si standard. Crystallinity was calculated as the ratio of total scattering from α-PLLA relative to the amorphous hump. The amorphous contribution was determined in two different ways. First, the classical relative crystallinity index was determined by (1) subtracting background scattering with a quasi-linear baseline (2) tracking a polynomial baseline at the base of the peaks to separate crystalline from amorphous contribution and (3) calculating area-based crystalline fraction. In the second approach, the amorphous fraction was modelled with a Pawley single or multiple peaks, as the deconvolution required, and from the obtained crystalline to amorphous ratio, the crystallinity percentage was determined. Although this second approach is not commonly used in polymer XRD, we have found that it could give more accurate results, since it incorporates crystal structure-based peak broadening due to crystallite size, thus it accounts for a crystalline fraction in the border of the microcrystalline-amorphous region. Additionally, this method allows for the calculation of the average crystallite size and distribution. 

The chemical structure of the PLLA samples was analysed using Fourier-transform infrared spectroscopy (FTIR, Vertex 70 spectrometer, Bruker, Billerica, MA, USA) in the wavenumber range of 4000 to 400 cm^−1^. The measurements were operated by averaging 50 scans and a resolution of 4 cm^−1^. 

Polarised transmitted light microscopy was applied to test the microscale crystallinity of samples. Lamellae of ~0.1 mm in thickness were cut from the test pieces and mounted on a glass plate without glue. A Zeiss AxioLab Imager A2m microscope with 3200K white source and AxioCam MRc5 digital camera was used to capture micrographs at crossed Nicole.

The surface morphology of the PLLA samples was verified by scanning electron microscopy (SEM). SEM measurements were done with a Thermo Scientific Helios G4 PFIB CXe and JEOL JSM 7200F instruments. Before the measurement, the samples were mounted on a conductive carbon tape and these were coated with a thin Au/Pd layer in Ar atmosphere. 

## 4. Results and Discussion

### 4.1. Determination of Solubility Parameter (δ) of PLLA

Table 1 contains the molar attraction constants ((cal cm^3^)^1/2^ mol^−1^) and molar mass (g/mol) of various groups of PLLA monomer or repeating unit necessary for the calculation of solubility parameter of PLLA by Small’s method. The density of PLLA is 1.32 g/cm^3^, therefore, the solubility parameter, δ = (ΣF)/V = (ΣF)ρ/M = (552 × 1.32)/72 = 10.12 cal^1/2^cm^−3/2^ = (10.12 × 2.045) MPa^1/2^ = 20.7 MPa^1/2^. By using Small’s method, the solubility parameter of the PLLA sheet was found to be 20.7 MPa^1/2^.

**Figure 2:**
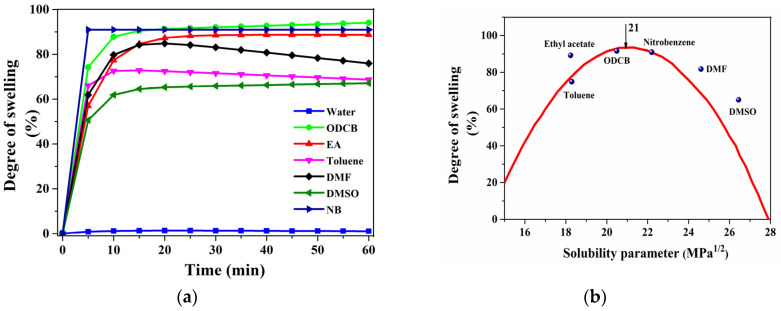
Saturation curve of PLLA in various organic solvents (**a**); Degree of swelling (%) Vs solubility parameter of various solvents (MPa^1/2^) (**b**).

The software slide-write plus for Windows [31] was used to fit the swelling (%) Vs time (min) curve. The equations used for curve fitting are a0(1 − exp(−x/a1)) and a0(1 − exp(−x/a1)) − a2x. Figure 2a represents the swelling behaviour and the time dependence of PLLA in various organic solvents and the saturation of solvent diffusion into the PLLA samples has been shown by the equilibrium degree of swelling of the polymeric network. The decreasing trend in the degree of swelling in case of solvents toluene and DMF is due to the disintegration and dissolution of gelatinous PLLA samples after 15 min. Figure 2b represents the solubility curve to determine the solubility parameter of PLLA. The solubility parameter of the solvents, the degree of swelling (%) of PLLA in the respective solvents and the calculation of (δ_p_ − δ_solv_)^2^, where δ_p_ and δ_solv_ are the solubility parameters of polymers and solvents, respectively, are shown in Table 2. Using the curve (Figure 2b), the solubility parameter of PLLA was found to be 21 MPa^1/2^. Certainly, the solubility parameter of PLLA determined from the experiment and small’s method is in good agreement.

It is familiar that liquids with similar solubility parameters are likely to be miscible and in the same way, polymers can dissolve in solvents with solubility parameter not too different from them. However, Gee [32] has shown a similar relationship between the swelling of polymers in solvents and their solubility parameters; i.e., swelling is maximal when (δ_p_ − δ_solv_)^2^ is zero. Our results of (δ_p_ − δ_solv_)^2^ (shown in Table 2) are quite in line with Gee’s interpretation. 

The mechanism for the solvent-induced crystallization involves two main sequential processes: 1. diffusion of the low molecular weight solvent into the interior of the polymer network (swelling), 2. segmental relaxation and polymer chain orientation (crystallization). It is well-known that the interaction between the polymer chains and the solvent molecules affects the solvent-induced crystallization of the polymer. Thus, the solvents with similar solubility parameter are important not only in polymer dissolution, but also have a significant influence in swelling and crystallization. These interactions are not only relying on the total solubility parameter, but rather on the individual three components such as dispersion force (δ_d_), dipolar intermolecular force (δ_p_) and hydrogen bonding (δ_h_), proposed by C. M. Hansen [26]. Though the solvents ethyl acetate and toluene share the same solubility parameter value, the degree of swelling of PLLA by toluene is lower than ethyl acetate. This can be explained by considering the individual components of SP, i.e., the dipolar intermolecular force and the hydrogen bonding parameter of toluene, which is relatively lower, resulted in weaker interaction with PLLA molecules when compared to ethyl acetate. The solubility parameter of the raw PLLA is found to be about 21 MPa^1/2^, which is similar to the values predicted in earlier literature [33]. As the solubility parameter of PLLA and the solvents nitrobenzene, o-dichlorobenzene and ethyl acetate (Table 2) with a high swelling behaviour (~ 90%) are nearly equal, the interaction between the PLLA molecular chain and the low molecular weight solvents is strong. Therefore, these three polar aprotic solvents with similar solubility parameters to PLLA were chosen for investigating the solvent-induced crystallization of PLLA at varying temperatures and treatment times. 

### 4.2. Crystallization Behaviour of PLLA Induced by Solvents

**Figure 3:**
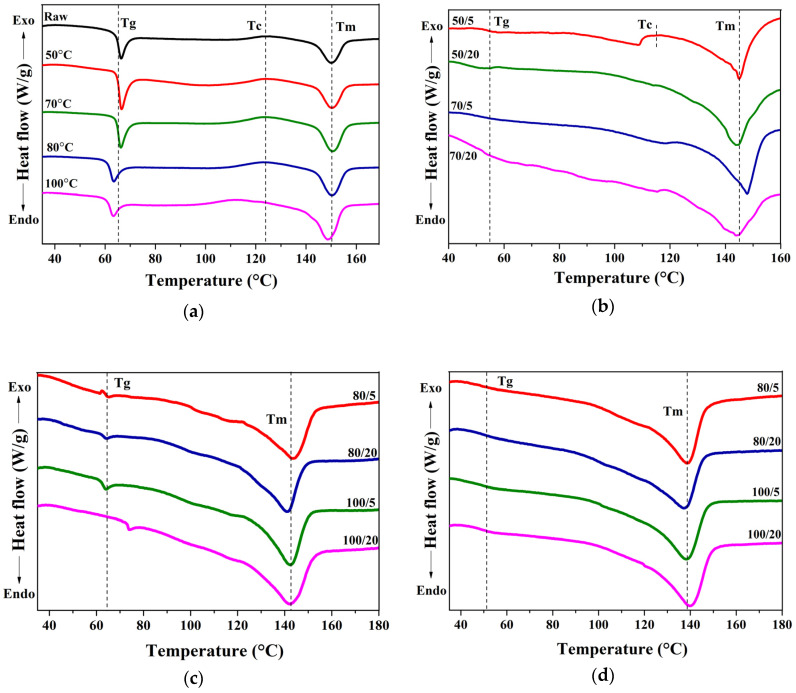
Differential scanning calorimetry (DSC) thermograms of PLLA; raw and thermally-annealed at different temperatures for 20 min (**a**); immersed in ethyl acetate at 50 and 70 °C (5, 20 min) (**b**); immersed in o-dichlorobenzene (ODCB) at 80 and 100 °C (5, 20 min) (**c**); immersed in nitrobenzene at 80 and 100 °C (5, 20 min) (**d**).

The crystallization behaviour of PLLA sheets induced by the solvents at different temperature and time is discussed using DSC thermogram. The changes in the DSC thermogram of the thermally-annealed and solvent-treated PLLA sheets at varying temperature and time are shown in Figure 3a–d.

The *T*_g_, *T*_c_, *T*_m_, Δ*H*_c_, Δ*H*_m_ and %X_c-DSC_ determined using DSC thermograms are summarized in Table 3 and Table 4. As the DSC data were used to determine the crystallinity, the first heat scan data are better, relative to second or third heat scans, since the first heat scan is the representation of the polymer structure as a direct result of the solvent supported heat treatment. For every PLLA sheet annealed at different temperatures and times, a sharp endothermic peak was observed at the glass transition temperature, *T*_g_ (59.0–65.2 °C). The broad exothermic peak between 112.2–125.0 °C, which indicates the crystallization of the PLLA samples and a melting peak around 150 °C were observed for all the annealed samples. As can be seen from Table 3, there is only a slight decrease in the glass transition temperature and not much change in the melting temperature of thermally annealed PLLA. The raw PLLA sheet taken for the experiment is semi-crystalline, with about 10.2% crystallinity. The percentage of crystallinity calculated using re-crystallization and melting enthalpies after annealing at 50–80 °C was relatively low and a negligible increase in crystallinity was observed for samples annealed at 100 °C. Based on the literature [34], the temperature taken for the cold-crystallization of PLLA is sufficient to form crystallites, but the lack of crystallization is due to the inadequate treatment time or the cooling rate required to form crystallites.

For solvent-treated PLLA samples (Table 4), we could observe a decrease in *T*_g_ for samples immersed in the solvent ethyl acetate (46.5–53.2 °C) and nitrobenzene (47.0–52.0 °C). The decrease in *T*_g_ is due to the increased mobility and segmental relaxation of the polymer chains (plasticization effect) during solvent treatment. On the other hand, the solvent ODCB did not much affect the *T*_g_ except at high temperature (100 °C) and longer treatment time (20 min). The increased *T*_g_ at 100 °C is observed in Figure 3c, since the higher molecular weight ODCB stiffens and restricts the mobility of the polymer chain, especially at longer treatment times. The absence of recrystallization peak and higher melting enthalpies represent the increased degree of crystallinity upon treatment with solvents. The melting peaks were shifted to a lower value, notably in ODCB (141–144 °C) and in nitrobenzene (137–140 °C). The decrease in melting endotherm indicates the presence of residual solvent molecules (as impurities) in the polymer crystallites, as the room temperature drying is not sufficient to evaporate higher boiling point solvents from the PLLA samples. The increase in the endothermic melting enthalpy in all the samples treated with solvents displayed the enhancement of crystallinity and the highest value was attained by the solvent nitrobenzene. 

**Figure 4:**
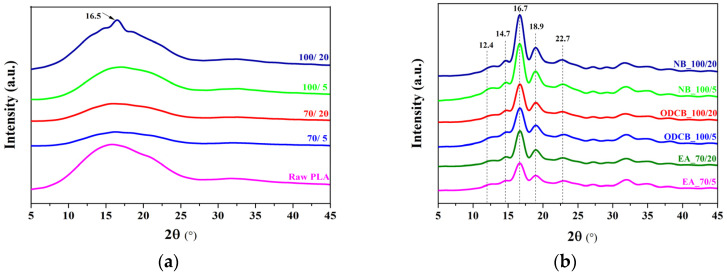
Wide-angle X-ray diffraction (WAXD) pattern of raw and thermally-annealed PLLA at different treatment temperatures (70 and 100 °C) and times (5 and 20 min) (**a**); PLLA crystallized in solvents at different treatment temperatures (70 and 100 °C) and times (5 and 20 min) (**b**).

The occurrence of crystallization induced by solvents at different temperatures and treatment times was further confirmed by XRD. The diffraction patterns of thermally annealed PLLA and samples crystallized in the solvents at different temperatures and treatment times are shown in Figure 4a,b.

The three major humps on the raw PLLA pattern show short molecular clusters corresponding to 2-3 molecules (~15° 2*θ* denoting ~6 Å), single molecules (~31.5° 2*θ* denoting ~3 Å) and the short chains laterally bridged by ~2 Å bond distance (~40.5° 2*θ*), assuming the molecular size of ~3 × 4 Å of the base material. In general, as a function of temperature, the PLLA can form different crystal structures such as α’ below 120 °C and α-crystal above 120 °C [35]. As can be seen from Figure 4a, we couldn’t observe any discreet peaks in the XRD pattern of thermally annealed PLLA samples, but there is a small rise in the peak (2*θ* = 16.5°) of PLLA samples annealed at 100 °C for 20 min. From the pattern profiles, compared to the raw PLLA, it is evident that the temperature chosen for thermal annealing (<120 °C) is sufficient for the segmental motion of the polymer chain and formation of α’ crystals, but as mentioned earlier, the treatment time or the cooling rate of the process was not enough to form crystallites during thermal annealing. Meanwhile, in the case of solvent-treated PLLA samples (Figure 4b), various diffraction peaks were observed at 2*θ* = 12.4°, 14.7°, 16.7°, 18.9° and 22.7°, corresponding to the sets of crystallographic planes (004/103), (010), (200/110), (203) and (015), respectively. It can be seen that the crystals formed in PLLA due to the polymer chain segmental relaxation created by the solvents, exhibited the more stable α-crystal polymorph, which is confirmed by the characteristic diffraction patterns of α-crystalline PLLA and the absence of a distinctive peak at 2*θ* = 24.4°, corresponding to α’-form [36]. The two distorted chains of α-structure of PLLA adopt a 10_3_ conformation and are packed in an orthorhombic unit cell of space group P2_1_2_1_2_1_ with lattice parameters a, b and c (given in Table 5), almost in line with the literature value (a = 10.683 Å, b = 6.170 Å and c = 28.860 Å) [30]. The effect of different solvents and curing times are reflected by the measured XRD peak and these differences are best shown by the unit cell size. The largest variation is observed along the c-axis, the chain length direction of the polymer fibres, indicating shorter bonds, therefore better conformation of α-crystal in PLLA after NB and ODCB treatment. The percentage of crystallinity calculated by the relative crystallinity index of XRD shows closer values to that of DSC results and very large differences are observed for the LeBail deconvolution method of XRD. These differences are attributed to a large number of crystallites closer to the amorphous boundary. In the case of raw and untreated PLLA, there are 2 unit cells along the c-axis, 4 unit cells along the a-axis and 10 unit cells along the b-axis (see Table 5 for crystallite size and the respective unit cell metrics) resulting in fibrous nanocrystals. After solvent treatment, the crystallite sizes increase, therefore the fraction of very small crystallites also increases. These nanometre-sized crystallites will significantly contribute to diffraction producing broad peaks. However, during DSC analysis, these crystallites undergo further crystal size growth and thus behave like an amorphous component.

**Figure 5:**
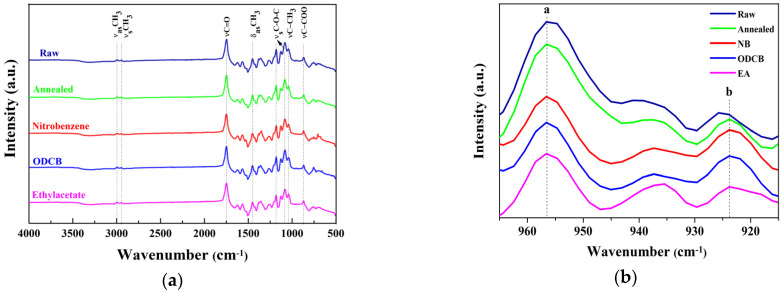
FTIR spectra of raw, annealed and solvent treated PLLA (at 100 °C, 20min) (**a**); Enlarged FTIR spectra of 5a curve in the range 965–915 cm^−1^ (**b**).

The changes in the infrared spectra of the PLLA sheets induced by thermal-annealing and solvents at 100 °C for 20 min are shown in Figure 5a,b. The FTIR spectra showed distinctive absorption bands assigned to their different functional groups, indicating the peaks at 2997 cm^−1^ and 2946 cm^−1^ to the asymmetric and symmetric stretching vibration of C–H_3_ group, respectively, in the PLLA polymer chain. The major spectral band at 1749 cm^−1^ (assigned to tt conformers) represents the carbonyl stretching vibration and the band at 1458 cm^−1^ aroused from the asymmetric deformation vibration of C–H_3_ [37]. Furthermore, the absorption bands at 1187 and 1089 cm^−1^ are due to the symmetric stretching vibration of C–O–C and the band at 871 cm^−1^ representing the vibration of the C–COO [38]. There is no disappearance or shift of the characteristic band at 1749 cm^−1^ indicative of α-crystals [35]. The fingerprint regions play a crucial role in determining the crystallinity of polylactides. From Figure 5b, we could observe a band at 956 cm^−1^ in all the samples, indicating the amorphous phase of PLLA samples and during crystallization, the intensity of this band decreases [39]. On the other hand, a small band appears around 923 cm^−1^ attributed to the coupling of C–C backbone stretching with the CH_3_ rocking mode. This band is sensitive to the 10_3_ helix chain conformation of PLLA α crystals, hence, it is called the crystalline band of PLLA [40]. Since the raw PLLA contains a negligible crystallinity, the band at 923 cm^−1^ is also present in the raw sample. On the other hand, we can find a notable decrease in the intensity of the peaks at 956 cm^−1^ and an increase in the intensity of the peaks at 923 cm^−1^, especially for the samples immersed in solvents, which again confirmed the solvent-induced crystallization of PLLA. 

### 4.3. Crystal Morphological Evaluation of PLLA

**Figure 6:**
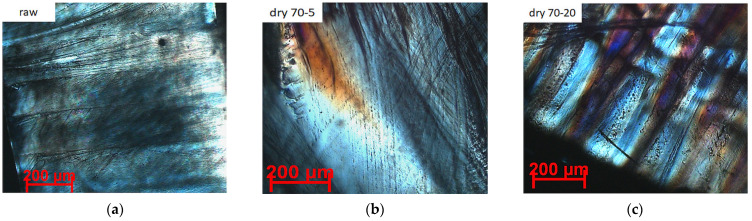

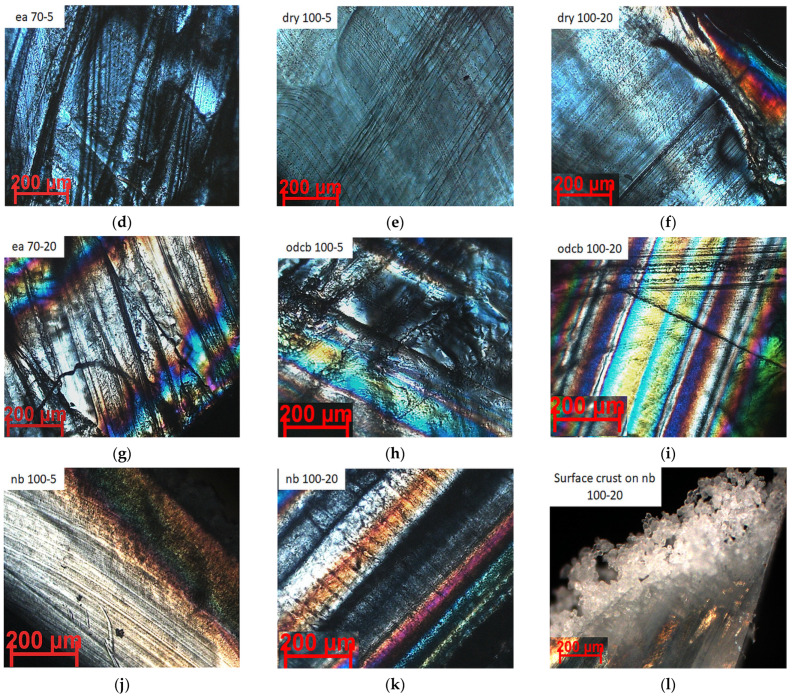
Polarised optical micrograph (POM) of raw PLLA (**a**); PLLA annealed at 70 °C for 5 min (**b**); PLLA annealed at 70 °C for 20 min (**c**); PLLA crystallized in ethyl acetate at 70 °C for 5 min (**d**); PLLA annealed at 100 °C for 5 min (**e**); PLLA annealed at 100 °C for 20 min (**f**); PLLA crystallized in ethyl acetate at 70 °C for 20 min (**g**); PLLA crystallized in ODCB at 100 °C for 5 min (**h**); PLLA crystallized in ODCB at 100 °C for 20 min (**i**); PLLA crystallized in nitrobenzene at 100 °C for 5 min (**j**); PLLA crystallized in nitrobenzene at 100 °C for 20 min (**k**,**l**).

The main scope of the polarised optical microscopy (POM) investigations was to observe the crystallinity through the presence of interference colours and extinction on the different PLLA samples. Images were recorded in the maximum illumination position with the texture visible, on the thinnest (<100 μm) edges of the samples. In most of the samples, the fibrous texture is observed as nanofiber bundles of cca. 1 μm. The optically anisotropic character of the crystals formed from the solvent-induced crystallization of PLLA is given in Figure 6a–l. Compared to the raw PLLA sheet, a layered structure was developed by solvent-induction and heat treatment, as a result of polymer chain rearrangement and crystallization [22]. The appearance and the intensity of interference colours are in agreement with the XRD and DSC data i.e., the larger crystallites and crystallinity was achieved for the specimens with well-developed layers and interference colours. This observation further indicates that the crystallinity calculated by DSC and the relative crystallinity by XRD is underestimating the actual crystallinity, since the values obtained by LeBail deconvolution are closer to a fully crystallized polymer body. Moreover, a spheroidal-globular crust has been developed on the surface of the test specimens, which is amorphous in composition, with a high refractive index. This is probably a dissolution product of the PLLA in solvents. In the middle of the sample, the interference colours can be visible, whereas only the intense reflectivity of the material could be observed on the surface, as shown in Figure 6l.

SEM investigation was done to understand the surface morphology and surface roughness of the PLLA sheets crystallized in various solvents. Different crystal morphologies were obtained for each solvent, as the nucleation and crystal growth due to solvent-induced crystallization of PLLA depends on various factors, such as solvent evaporation rate, the interfacial tension between the polymer and the solvent, viscosity, etc [41].

**Figure 7:**
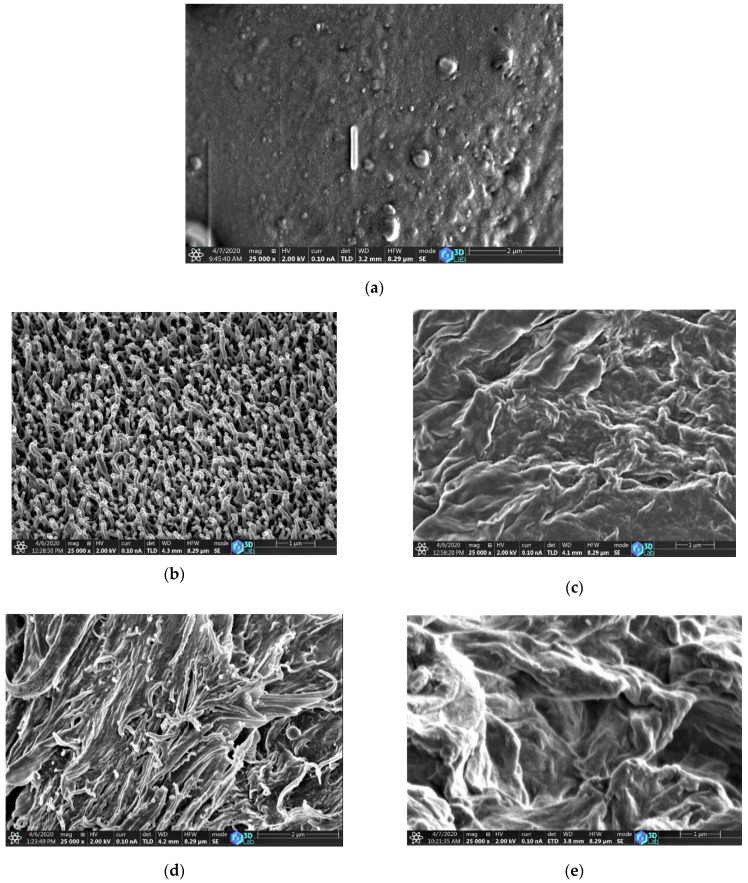
SEM micrograph of raw PLLA (**a**); PLLA crystallized in ethyl acetate at 70 °C for 20 min (**b**); nitrobenzene at 100 °C for 20 min (**c**); ODCB at 100 °C for 5 min (**d**); ODCB at 100 °C for 20 min (**e**).

Here, we could observe a difference in morphology while using solvents with different boiling temperatures, i.e., the lower boiling point solvent ethyl acetate evaporates faster from the crystal growth front of the PLLA sheet, therefore the nucleation process dominates the crystal growth, leading to the formation of small uniform rod-like lamellae morphology, which is shown in the cross-sectional view of the SEM micrograph (Figure 7b). On the other hand, the other two solvents, having a high boiling point, reside inside the polymer matrix for a long time, owing to the slower evaporation rate of the solvent, resulting in producing larger crystals with different morphologies at varying temperatures and treatment times, as shown in Figure 7c–e.

## 5. Conclusions

The solvent-induced crystallization of PLLA sheet was investigated using different thermodynamically compatible organic solvents. Since the solubility parameter of PLLA and the solvents ODCB and nitrobenzene are relatively similar, the interaction between the amorphous PLLA chain and the solvents is quite intense. This facilitates the smooth diffusion of solvent molecules into the PLLA matrix, which stimulates the random movement of the polymer molecular chain, thereby inducing the orderly arrangement of chains with a high degree of crystallinity. The effect of different solvents at varying temperatures and curing times on the crystallization of PLLA sheet was explained using different analysing techniques. The differences were shown perfectly by the unit cell size, especially along the chain length direction of the polymer fibres, indicating shorter bonds, therefore a better conformation of α-crystals during NB and ODCB treatment. The percentage of crystallinity calculated using the classical relative crystallinity index of XRD shows closer values to that of DSC, but a large variation is observed while using the LeBail deconvolution method. These differences are attributed to the presence of a large number of the crystallites near the amorphous boundary. However, the LeBail deconvolution method gives more accurate results, as it includes crystal structure-based peak broadening due to crystallite size, thus, it accounts for a crystalline fraction in the border of the microcrystalline-amorphous region. Moreover, the appearance of strong birefringence in the polarised optical micrograph and the different crystal morphologies of the SEM micrograph indicates the orientation of larger crystallites and a high degree of crystallinity developed by the combined effect of solvent-induction and heat treatment of the PLLA.

## Figures and Tables

**Table 1:** Determination of solubility parameter of PLLA.

Group	Number (n)	Molar Attraction, F (cal cm^3^)^1/2^ mol^−1^	Fxn (cal cm^3^)^1/2^ mol^−1^	Molar Mass (g/mol)
–CH_3_	1	214	214	15
>CH-	1	28	28	13
–COO–	1	310	310	44
**Σ**			**552**	**72**

**Table 2:** Data of solubility parameter of solvents and the degree of swelling of PLLA.

Solvents	Solubility Parameter (MPa^1/2^) [25,26]	(δ_p_^1^ − δ_solv_)^2^ [32]	Swelling (%)
Toluene	18.2	7.8	74.8
Ethyl acetate	18.2	7.8	89.0
ODCB	20.5	0.3	91.5
nitrobenzene	22.2	1.4	91.0
DMF	24.9	15.2	82.0
DMSO	26.7	32.5	65.4
Water	48.0	729.0	1.6

^1^ The solubility parameter of the PLLA sheet, δ_p_ = 21 MPa^1/2^ calculated from the swelling experiment is used in the table.

**Table 3:** DSC data of raw and annealed PLLA sheet.

Sample	*T*_g_ (°C)	*T*_c_ (°C)	*T*_m_ (°C)	Δ*H*_c_ (J/g)	Δ*H*_m_ (J/g)	%X_c_
Raw polylactide (PLLA)	65.3	124.1	150.0	2.6	12.1	10.2
50/5	59.0	118.8	148.8	7.6	14.5	7.4
50/20	59.9	125.0	150.9	3.8	11.1	7.8
70/5	65.0	124.8	150.6	3.1	11.4	8.9
70/20	65.2	123.5	150.6	5.3	13.8	9.1
80/5	63.6	124.7	151.5	2.6	10.5	8.5
80/20	62.4	123.3	150.4	5.2	13.9	9.3
100/5	62.4	124.2	150.1	2.9	16.6	14.7
100/20	62.3	112.2	148.7	6.1	21.1	16.1

**Table 4:** DSC data of solvent treated PLLA.

Sample	*T*_g_ (°C)	*T*_c_ (°C)	*T*_m_ (°C)	Δ*H*_c_ (J/g)	Δ*H*_m_ (J/g)	%X_c_
EA_50/5	52.7	114	145	2.0	19.7	19.0
EA_50/20	46.5	-	144.2	0	29.2	31.4
EA_70/5	53.2	-	147.8	0	24.2	26.0
EA_70/20	52.8	-	144.4	0	26.6	28.6
ODCB_80/5	63.7	-	143.6	0	27.6	29.6
ODCB_80/20	62.8	-	141.1	0	27.0	29.0
ODCB_100/5	62.5	-	142.3	0	26.4	28.4
ODCB_100/20	73.5	-	142.2	0	31.0	33.3
NB_80/5	47.5	-	138.5	0	34.6	37.2
NB_80/20	52.0	-	137.4	0	35.8	38.5
NB_100/5	50.2	-	138.3	0	32.9	35.3
NB_100/20	47.0	-	139.8	0	35.5	38.1

**Table 5:** Degree of crystallinity, crystallite size and lattice parameters calculated from XRD data.

Sample	Crystallinity (%)			Crystallite Size (nm)	Lattice Parameters (Å)		
	DSC ^1^	rel.c. ^2^	LeBail ^3^		a	b	c
Theoretical [30]	-	-	-	-	10.683	6.170	28.860
Raw PLA	10.2	10.4	22.7	4 ± 1	10.125	5.927	31.345
Annealed_70 ℃/5 min	8.9	9.4	12.9	4 ± 1	9.986	5.911	32.044
Annealed_70 ℃/20 min	9.1	11.1	25.5	4 ± 1	10.050	5.914	31.953
Annealed_100 ℃/5 min	14.7	12.5	18.8	4 ± 1	10.124	5.922	31.353
Annealed_100 ℃/20 min	16.1	14.1	15.6	11 ± 3	10.025	5.957	31.600
EA_50 °C/20 min	31.4	20.9	50.8	18 ± 4	9.790	6.021	31.608
EA_70 ℃/5 min	26.0	30.0	67.4	13 ± 3	9.833	6.026	31.714
EA_70 ℃/20 min	28.6	36.4	60.3	15 ± 4	9.785	6.036	31.686
ODCB_100 ℃/5 min	28.4	26.0	80.0	8 ± 2	10.653	6.096	28.845
ODCB_100 ℃/20 min	33.3	30.4	81.4	10 ± 2	10.628	6.099	28.778
NB_100 ℃/5 min	35.3	33.8	77.8	11 ± 3	10.638	6.115	28.804
NB_100 ℃/20 min	38.1	34.5	84.4	12 ± 3	10.642	6.114	28.801

^1^ Crystallinity (%) calculated from DSC data. ^2^ rel.c. is the crystallinity (%) calculated using the classical relative crystallinity index of XRD. ^3^ LeBail is the crystallinity (%) calculated using the LeBail deconvolution method of XRD.
